# Prediction of genetic merit for growth rate in pigs using animal models with indirect genetic effects and genomic information

**DOI:** 10.1186/s12711-020-00578-y

**Published:** 2020-10-07

**Authors:** Bjarke G. Poulsen, Birgitte Ask, Hanne M. Nielsen, Tage Ostersen, Ole F. Christensen

**Affiliations:** 1 Center for Quantitative Genetics and Genomics, Blichers Allé 20, 8830 Tjele, Denmark; 2 SEGES, Danish Pig Research Centre, Danish Agriculture and Food Council F.m.b.A., Axelborg, Axeltorv 3, 1609 Copenhagen V, Denmark

## Abstract

**Background:**

Several studies have found that the growth rate of a pig is influenced by the genetics of the group members (indirect genetic effects). Accounting for these indirect genetic effects in a selection program may increase genetic progress for growth rate. However, indirect genetic effects are small and difficult to predict accurately. Genomic information may increase the ability to predict indirect genetic effects. Thus, the objective of this study was to test whether including indirect genetic effects in the animal model increases the predictive performance when genetic effects are predicted with genomic relationships. In total, 11,255 pigs were phenotyped for average daily gain between 30 and 94 kg, and 10,995 of these pigs were genotyped. Two relationship matrices were used: a numerator relationship matrix (${\mathbf{A}}$) and a combined pedigree and genomic relationship matrix (${\mathbf{H}}$); and two different animal models were used: an animal model with only direct genetic effects and an animal model with both direct and indirect genetic effects. The predictive performance of the models was defined as the Pearson correlation between corrected phenotypes and predicted genetic levels. The predicted genetic level of a pig was either its direct genetic effect or the sum of its direct genetic effect and the indirect genetic effects of its group members (total genetic effect).

**Results:**

The highest predictive performance was achieved when total genetic effects were predicted with genomic information (21.2 vs. 14.7%). In general, the predictive performance was greater for total genetic effects than for direct genetic effects (0.1 to 0.5% greater; not statistically significant). Both types of genetic effects had greater predictive performance when they were predicted with ${\mathbf{H}}$ rather than ${\mathbf{A}}$ (5.9 to 6.3%). The difference between predictive performances of total genetic effects and direct genetic effects was smaller when ${\mathbf{H}}$ was used rather than ${\mathbf{A}}$.

**Conclusions:**

This study provides evidence that: (1) corrected phenotypes are better predicted with total genetic effects than with direct genetic effects only; (2) both direct genetic effects and indirect genetic effects are better predicted with ${\mathbf{H}}$ than ${\mathbf{A}}$; (3) using ${\mathbf{H}}$ rather than ${\mathbf{A}}$ primarily improves the predictive performance of direct genetic effects.

## Background

Quantitative geneticists are increasingly interested in estimating genetic parameters for the impact of an individual on the phenotypes of other individuals [1]. The expectation is that selection for such beneficial interactions between animals for a certain trait will increase genetic progress in that trait [2]. Pigs interact with one another and they have been subject to genetic analyses of interactions [3–5]. Since these interactions may give rise to previously unexploited heritable variation [6], inclusion of genetic effects of interactions between pigs in animal models for the estimation of breeding values may improve the efficiency of breeding programs [7].

Griffing [6] proposed an animal model for estimating simultaneously both an additive genetic effect on own performance (direct genetic effect) and an additive genetic effect on the performance of other individuals (indirect genetic effect) on the same trait. Using these models should enable the utilization of the heritable (co)variation that arises from these interactions [7]. In addition, the association between direct and indirect genetic effects may explain why some selection schemes result in unexpected responses, whether smaller or larger than expected [6–8].

Indirect genetic effects have proven challenging to quantify and predict. Some studies on growth rate in pigs show that including indirect genetic effects in the animal model improved its goodness-of-fit and/or its predictive performance [4, 9, 10], whereas other studies found neither of these results [3, 11, 12]. Some of these studies may have been challenged by both the complex nature of interactions between pigs and the fact that indirect genetic models are more sensitive to experimental design/data structure than classical animal models [9, 13–17]. A major challenge with predicting indirect genetic effects is that they are often small and thereby require more information for accurate prediction than direct genetic effects.

Modelling indirect genetic effects with genomic information may offer a solution to the abovementioned challenge since estimating genetic relationships between pigs using genomic information rather than pedigree information increases the accuracies of the coefficients of kinship. While the estimated genetic relationship from pedigree information depends on the number of generations that is traced [18], genomic information captures both signals of selection and drift from all previous generations [19]. Consequently, genomic information is better for tracing direct genetic effects than pedigree information and could be better for indirect genetic effects too.

For direct genetic effects, estimating the genetic relationships between pigs using genomic information rather than pedigree information may result in smaller estimated variance components [20]. This may have both analytical and genomic causes [21–24]. Therefore, it is not advised to estimate variance components using genomic information [24].

In spite of the challenges associated with parameter estimation, genomic information has proven very beneficial when predicting direct genetic effects [25–27] and can be expected to be beneficial when predicting indirect genetic effects as well. This is indicated in the study by Alemu et al. [28] that predicted the sums of direct and indirect genetic effects and found that genomic information improved the predictive performance. However, their study does not answer the question of whether genomic information increased the predictive performance of direct genetic effects, indirect genetic effects or both. To our knowledge, no study has investigated whether indirect genetic effects increase the predictive performance of animal models when direct and indirect genetic effects are predicted simultaneously with genomic information.

We hypothesize that: (1) the combined predictive performance of indirect and direct genetic effects is superior to the predictive performance of only direct effects; and (2) both prediction with the combination of indirect and direct genetic effects and prediction with direct genetic effects only is more accurate with genomic relationships than with pedigree relationships.

## Methods

In this section, we successively present (1) the phenotypic data; (2) the definitions of standardized starting weight covariates; (3) the characteristics, imputation, and quality control of the genotypic data; (4) the construction of relationship matrices with either pedigree information only or both pedigree and genomic information; (5) the linear mixed models with either both indirect- and direct- genetic effects or direct genetic effects only; (6) the equations to calculate variance components and heritability statistics; and (7) the procedure used to estimate the predictive performances of genetic levels from the linear mixed models.

No Animal Care and Use Committee approval was obtained for this study because the pigs were part of routine performance tests in the DanBred breeding program. All pigs were kept in accordance with both Danish legislation for pig production and the Danish Product Standard [29].

All data was provided by SEGES, Danish Pig Research Centre.

### Phenotypic data

The data was recorded in a DanBred Landrace nucleus herd in which pigs were performance-tested for average daily gain between August 2015 and October 2018. Boars and gilts were housed in different stables, and pigs were produced on-farm through artificial insemination. The test scheme was carried out on a weekly basis. The pigs entered the test when they weighed more than 28 kg with the restriction that their entry date could not be later than 2 weeks after the first pig in the pen entered the test. The test of a group ended when the largest pig in the pen reached 94 kg. The age at test start and number of days in test differed between pen-mates, since pigs within a group could enter the test at different dates. All pigs were weighed at the start and end of the test. Growth rate was defined as the average daily gain in live weight from start to end of the test; i.e. average daily gain is the average weight gain per day during the test period. Pigs that were removed from the pen before the end of the test obtained no measurement on daily gain, but were kept in the dataset (165 pigs). The final dataset contained 11,420 pigs (6422 boars and 4833 gilts) among which 11,255 pigs had an average daily gain measurement, 1179 dams of phenotyped animals, 384 sires of phenotyped animals, 1197 groups (boar groups: 646; gilts groups: 551), and 1595 litters.

### Group structure

Pigs were allocated to groups based on liveweights prior to test start: i.e. heavy pigs were grouped with other heavy pigs and vice versa, which means that relatedness between group members was not considered when grouping the animals. The group size at test start was 9 for gilts and 10 for boars. The average group size was 9.57 pigs at test start because there were more groups with boars than with gilts. Due to removals, a pig had on average 8.5 group members per day. Groups contained pigs from on average 7.7 litters (min: 4; max 10; SD: 1.2) and 6.6 sires (min: 3; max 10; SD: 1.3). The average relatedness among group members is presented as part of the results.

### Definition of covariables

Two covariables related to starting weight were defined: ${\text{sWghtAge}}$ and ${\text{WghtDev}}$.

${\text{sWghtAge}}$ is the expected weight of pigs at the average age pigs were weighed. The growth rate of a pig is approximately a sigmoid function of the age (developmental stage) of the pig [30]. Thus, the observed growth rate depends on the developmental stage at which it is measured. The developmental stage of a pig depends primarily on its age and previous feed intake [31]. The pigs in this study differed in developmental stages, in particular due to variation in the age at which they entered the test (Table 1). In an attempt to account for these differences in developmental stage of the pigs in the animal model, starting weights were corrected for the age at which they were observed:

1 $${\text{sWghtAge}}_{\text{i}} = {\text{sWght}}_{\text{i}} - \frac{{\text{sWght}_{\text{i}} }}{\text{sAge}}_{\text{i}} \left( {\text{sAge}_{\text{i}} - {\overline{\text{sAge}}}} \right),$$

where ${\text{sWghtAge}}_{\text{i}}$ is the weight at test start corrected for difference in age, ${\text{sWght}}_{\text{i}}$ is the observed weight at test start in kg, ${\text{sAge}}_{\text{i}}$ is the age at test start in days, and ${\overline{\text{sAge}}}$ is the average age in days at test start across all animals.

**Table 1:** Mean, standard deviation, minimum and maximum of the phenotype and covariates

Variable	Mean	SD	Min	Max
Average daily gain (g/day)	1117.8	152.7	90.9	1580. 0
${\text{sWghtAge}}$ (kg)	29.76	2.87	16.20	41.13
${\text{sWghtDev}}$ (kg)	0.01	2.45	− 11.94	9.53
Average number of group members in the group (heads/day)	8.5	0.6	6.2	9.0

SD, Standard deviation; ${\text{sWghtAge}}$, Starting weight adjusted for age at start; ${\text{sWghtDev}}$, Deviation in date-adjusted starting weight from group mean

${\text{WghtDev}}$ is the expected deviation in start weight from the group mean at the average date the group was weighed. If a pig is lighter than its group members, it is at a disadvantage when competing for resources. To calculate ${\text{WghtDev}}$, first we standardized starting weights to one timepoint within each group:

2 $${\text{sWghtDate}}_{\text{i}} = {\text{sWght}}_{\text{i}} - \frac{{{\text{sWght}}_{\text{i}} }}{{{\text{sAge}}_{\text{i}} }}\left( {{\text{sDate}}_{\text{i}} - {\overline{\text{sDate}}}_{\text{g}} } \right)$$

where ${\text{sAge}}$ is as defined for ${\text{sWghtAge}}$; ${\text{sDate}}_{\text{i}}$ is the date at which the starting weight was observed for pig i; and ${\overline{\text{sDate}}}_{\text{g}}$ is the average date at which the starting weight was observed in group ${\text{g}}$. Lastly, ${\text{WghtDev}}$ was defined as the difference in ${\text{WghtDate}}$ from the group mean:

3 $${\text{sWghtDev}}_{\text{i}} = {\text{sWghtDate}}_{\text{i}} - {\overline{\text{sWghtDate}}}_{\text{g}},$$

where ${\overline{\text{sWghtDate}}}_{\text{g}}$ is the average ${\text{sWghtDate}}$ within group ${\text{g}}$. The mean, standard deviation, minimum, and maximum of ${\text{sWghtAge}}$ and ${\text{sWghtDev}}$ are in Table 1.

### Genotypic data and quality control

Pigs were genotyped with the NEOGEN GeneSeek^®^ Genomic Profiler Porcine BeadChip [32]. This chip provides information on genotypes on both autosomal and sex chromosomes. The aim was to genotype all the pigs, but due to sampling errors, only 10,998 pigs (96%) were genotyped.

Genotypes from three animals that failed DanBred’s parentage tests were omitted. Genotypes were regarded as valid if their GenCall-scores (GC-score) were above 60% [33]. Quality control of the genotypic data was performed per single nucleotide polymorphism (SNP) only. A SNP was used in later steps of the analysis if its call-rate was greater than 90%; its minor allele frequency was greater than 1%; its frequency of Mendelian errors was less than 0.1%; and its *p* value for the test of Hardy–Weinberg equilibrium was greater than 10^−7^. The Hardy–Weinberg criterion was not applied to SNPs on the sex chromosomes. After quality control of SNPs, missing genotypes were imputed with the FImpute v2.2 software [34]. In total, 34,123 SNPs and genomic information from 10,995 pigs was used for further analysis.

### Relationship matrices

Genetic effects were predicted with two types of relationship matrices: a numerator relationship matrix (${\mathbf{A}}$), and a combined pedigree and genomic relationship matrix (single-step; ${\mathbf{H}}$). ${\mathbf{A}}$ was constructed with a pedigree containing ancestors five generations prior to pigs with phenotypes. ${\mathbf{H}}$ was constructed from ${\mathbf{A}}$ and a genomic relationship matrix (${\mathbf{G}}$) using the single-step method [26, 35]. ${\mathbf{G}}$ was constructed using Method 1 of VanRaden [36] with centering and scaling based on the allelic frequencies observed for all genotyped pigs in the pedigree. The diagonal and off-diagonal elements of ${\mathbf{G}}$ were scaled and centered to have the same mean as the part of ${\mathbf{A}}$ that relates to genotyped animals [37–40]. Lastly, ${\mathbf{G}}$ was replaced by the weighted average between ${\mathbf{G}}$ and ${\mathbf{A}}$ to take into account that marker genotypes do not capture all the genetic variance:

4 $${\mathbf{G}}_{\text{w}} = {\text{w}}{\mathbf{G}} + \left( {1 - {\text{w}}} \right){\mathbf{A}},$$

where ${\text{w}}$ is the weight on the genomic relationship matrix. The value of ${\text{w}}$ ranged from 0 to 99% across prediction analyses (see section on “Predictive performance”).

### Animal models

Growth rate was analyzed with two linear mixed models: an animal model with both direct and indirect genetic effects (INDIRECT) and a classical animal model with only direct genetic effects (CLASSIC). For both models, variance components were estimated with ${\mathbf{A}}$ as covariance structure for genetic effects. Estimated variance components will be referred to by using the names of the models (DIRECT/INDIRECT). Variance component estimates and their respective model were then used to predict genetic effects with either ${\mathbf{A}}$ or ${\mathbf{H}}$ as covariance structure for genetic effects. In the following, prediction of genetic effects using CLASSIC and ${\mathbf{A}}$ as covariance structure will be referred to as CLASSIC_PED, prediction of genetic effects using CLASSIC and ${\mathbf{H}}$ as covariance structure will be referred to as CLASSIC_GEN, prediction of genetic effects using INDIRECT and ${\mathbf{A}}$ as covariance structure will be referred to as INDIRECT_PED, and prediction of genetic effects using INDIRECT and ${\mathbf{H}}$ as covariance structure will be referred to as INDIRECT_GEN.

Variance components were estimated with the average information restricted maximum likelihood (AI-REML) algorithm and genetic effects were predicted with best linear unbiased prediction (BLUP). Both the estimation of variance components and BLUP were carried out with the DMU software [41].

INDIRECT, the model with both indirect and direct genetic effects is as follows:

5 $${\mathbf{y}} = {\mathbf{Xb}} + {\mathbf{Z}}_{\text{D}} {\mathbf{a}}_{\text{D}} + {\mathbf{Z}}_{\text{I}} {\mathbf{a}}_{\text{I}} + {\mathbf{Wu}} + {\mathbf{Q}}_{\text{D}} {\mathbf{l}}_{\text{D}} + {\mathbf{Q}}_{\text{I}} {\mathbf{l}}_{\text{I}} + {\mathbf{R}}_{\text{I}} {\mathbf{e}}_{\text{I}} + {\mathbf{e}}_{\text{D}},$$

where subscripts ${\text{D}}$ and ${\text{I}}$ indicate whether an effect is direct or indirect, and the absence of a subscript indicates that the effect is expected to capture both direct and indirect effects; ${\mathbf{y}}$ is a vector of growth rates during the performance test; ${\mathbf{b}}$ is a vector of parameters for the fixed effects. The fixed class effects are sex and the interaction level between year of birth and month of birth. The fixed continuous effects are starting weight (${\text{sWghtAge}}$; see Eq. (2)), deviation in starting weight from the group mean (${\text{sWghtDev}}$; see Eqs. (3) and (4)), and the average number of group members present in the pen during the performance test (heads/day). The latter is used to correct for the effect of stocking density as pigs were removed during the test; ${\mathbf{a}}_{\text{D}}$ is a vector of direct genetic effects; ${\mathbf{a}}_{\text{I}}$ is a vector of indirect genetic effects; ${\mathbf{u}}$ is a vector of sex-year-month effects; ${\mathbf{l}}_{\text{D}}$ is a vector of direct litter effects; ${\mathbf{l}}_{\text{I}}$ is a vector of indirect litter effects, i.e. effect of litters of group mates; ${\mathbf{e}}_{\text{I}}$ is a vector of indirect environmental animal effects, i.e. this effect is analogous to a group effect; ${\mathbf{e}}_{\text{D}}$ is a vector of residuals; and ${\mathbf{X}}$, ${\mathbf{Z}}_{\text{D}}$, ${\mathbf{Z}}_{\text{I}}$, ${\mathbf{W}}$, ${\mathbf{Q}}_{\text{D}}$, ${\mathbf{Q}}_{\text{I}}$, and ${\mathbf{R}}_{\text{I}}$ are design matrices. Model INDIRECT contains two vectors of random genetic effects (direct and indirect) and five vectors of random, independent environmental effects (sex-year-month, direct litter, indirect litter, indirect environmental animal and residual), assumed to follow the distribution: 

where $\sigma _{{{\text{a}}_{\text{D}} }}^{2}$ is the direct genetic variance; $\sigma _{{{\text{a}}_{\text{I}} }}^{2}$ is the indirect genetic variance; $\sigma_{{{\text{a}}_{{{\text{D}},{\text{I}}}} }}$ is the covariance between direct and indirect genetic effects; ${\mathbf{C}}$ is the covariance structure for genetic effects, i.e. ${\mathbf{C}}$ is ${\mathbf{A}}$ for models INDIRECT and INDIRECT_PED, and ${\mathbf{H}}$ for model INDIRECT_GEN; $\sigma_{\text{u}}^{2}$ is the sex-year-month variance; $\sigma_{{{\text{l}}_{\text{D}} }}^{2}$ is the direct litter variance; $\sigma_{{{\text{l}}_{\text{I}} }}^{2}$ is the indirect litter variance; $\sigma_{{{\text{e}}_{\text{I}} }}^{2}$ is the indirect non-genetic animal variance; $\sigma_{{{\text{e}}_{\text{D}} }}^{2}$ is the residual variance; and ${\mathbf{I}}_{\text{u}}$, ${\mathbf{I}}_{{{\text{l}}_{\text{D}} }}$, ${\mathbf{I}}_{{{\text{l}}_{\text{I}} }}$, ${\mathbf{I}}_{{{\text{e}}_{\text{I}} }}$, and ${\mathbf{I}}_{{{\text{e}}_{\text{D}} }}$ are identity matrices with dimensions equal to the number of levels of their respective effects.

CLASSIC, the model with only direct genetic effects, is as follows:

7 $${\mathbf{y}} = {\mathbf{Xb}} + {\mathbf{Z}}_{\text{D}} {\mathbf{a}}_{\text{D}} + {\mathbf{Wu}} + {\mathbf{Q}}_{\text{D}} {\mathbf{l}}_{\text{D}} + {\mathbf{Q}}_{\text{I}} {\mathbf{l}}_{\text{I}} + {\mathbf{R}}_{\text{I}} {\mathbf{e}}_{\text{I}} + {\mathbf{e}}_{\text{D}},$$

where matrix and vector notations are identical to those for INDIRECT (Eq. (5)). CLASSIC contains one vector of random genetic effects (direct) and five vectors of random, independent environmental effects (sex-year-month, direct litter, indirect litter, indirect environmental animal and residual), assumed to follow the distribution: 

where the notation is the same as for INDIRECT (Eq. (6)).

CLASSIC contains indirect litter and indirect non-genetic animal effects although it is meant to represent a usual animal model. The reasons are that these indirect effects explain phenotypic variation and that greater similarity between CLASSIC and INDIRECT makes the results on their log-likelihood-ratio test more related to whether indirect genetic effects exist or not.

A log-likelihood ratio test was used to test whether INDIRECT had a better model fit than CLASSIC: ${\text{P}}\left( {\Delta LogL} \right) = \chi ^{2} \left( { - 2\log_{\text{e}} \left[ {\frac{{\ell \left( {\text{CLASSIC}} \right)}}{{\ell \left( {\text{INDIRECT}} \right)}}} \right]} \right)$, where $\chi^{2} \left( \ldots \right)$ is the cumulative distribution function of the Chi square distribution with two degrees of freedom for the parameters $\sigma _{{{\text{a}}_{\text{I}} }}^{2}$ and $\sigma _{{{\text{a}}_{{{\text{D}},{\text{I}}}} }}$; $\ell \left( {\text{CLASSIC}} \right)$ is the likelihood value of CLASSIC; and $\ell \left( {\text{INDIRECT}} \right)$ is the likelihood value of INDIRECT.

### Variance components and heritability parameters

The equations used to calculate variance components and heritability statistics for both CLASSIC and INDIRECT are in Table 2. Two heritability statistics were calculated: direct heritability (${\text{h}}^{2}$) and total heritability (${\text{T}}^{2}$) [2].

**Table 2:** Definitions of variance components and heritability statistics

	Classic	Indirect
$\sigma_{TBV}^{2}$	None	$\sigma_{{a_{D} }}^{2} + 2\left( {\bar{n} - 1} \right)\sigma_{{a_{D,I} }} + \left( {\bar{n} - 1} \right)^{2} \sigma_{{a_{I} }}^{2}$
$\sigma_{env}^{2}$	$\sigma_{u}^{2} + \sigma_{{l_{D} }}^{2} + \left( {\bar{n} - 1} \right)\left( {\sigma_{{l_{I} }}^{2} + \sigma_{{e_{I} }}^{2} } \right) + \sigma_{{e_{D} }}^{2}$	
$\sigma_{p}^{2}$	$\sigma_{{a_{D} }}^{2} + \sigma_{env}^{2}$	$\sigma_{{a_{D} }}^{2} + \left( {\bar{n} - 1} \right)\sigma_{{a_{I} }}^{2} + r\left( {\bar{n} - 1} \right)\left( {2\sigma_{{a_{D,I} }} + \left( {\bar{n} - 2} \right)\sigma_{{a_{I} }}^{2} } \right) + \sigma_{env}^{2}$
$h^{2}$	$\sigma_{{a_{D} }}^{2} *\left[ {\sigma_{p}^{2} } \right]^{ - 1}$	
$T^{2}$	None	$\sigma_{TBV}^{2} *\left[ {\sigma_{p}^{2} } \right]^{ - 1}$

$\sigma _{\text{TBV}}^{2}$, total heritable variance.$\sigma_{{{\text{a}}_{\text{D}} }}^{2}$, direct genetic variance. $\bar{n}$, average group size at test start (9.57 pigs). $\sigma _{{{\text{a}}_{{{\text{I}},{\text{D}}}} }}$, direct–indirect genetic covariance. $\sigma _{{{\text{a}}_{\text{I}} }}^{2}$, indirect genetic variance. $\sigma_{\text{u}}^{2}$, sex-year-month variance. $\sigma _{{{\text{l}}_{\text{D}} }}^{2}$, direct litter variance. $\sigma _{{{\text{l}}_{\text{I}} }}^{2}$, indirect litter variance. $\sigma _{\text{e} _\text{I}}^{2}$, Indirect animal variance. $\sigma_{{{\text{e}}_{\text{D}} }}^{2}$, residual variance. $r$, average relatedness among animals in a pen, averaged across pens (16.6%). $\sigma _{\text{env}}^{2}$, total variance due to not-heritable variance components. $\sigma _{\text{P}}^{2}$: total phenotypic variance. ${\text{h}}^{2}$, direct heritability. ${\text{T}}^{2}$, total heritability

### Predictive performance

To estimate the predictive performance of the models, the data were divided into training data and validation data. Training data contained phenotypes on pigs in groups where all pigs were born before January 1st, 2018 (9431 pigs; 83.8%), whereas the validation data contained phenotypes on pigs not included in the training data (1824 pigs; 16.2%).

The predictive performance of the animal models was defined as the ability to use the training data to predict corrected phenotypes of pigs in the validation data. The predictive performance was quantified as Pearson’s correlation coefficient between corrected phenotypes and predicted genetic levels. Here, the corrected phenotype was defined as the difference between observed phenotypes and fixed effects from INDIRECT: ${\mathbf{y}}_{\text{c}} = {\mathbf{y}} - {\mathbf{Xb}}$. The predicted genetic level was either based on direct genetic effects only, ${\mathbf{DGE}}_{\text{i}} = {\mathbf{a}}_{\text{i}}^{\text{D}}$, or on a combination of direct and indirect genetic effects, ${\mathbf{TGE}}_{\text{i}} = {\mathbf{a}}_{\text{i}}^{\text{D}} + \mathop \sum \nolimits_{{{\text{j}} \ne {\text{i}}}}^{\text{n}} {\mathbf{a}}_{\text{j}}^{\text{I}}$ (total genetic effect), where ${\mathbf{a}}_{\text{i}}^{\text{D}}$ is the direct genetic effect of pig ${\text{i}}$, and ${\mathbf{a}}_{\text{j}}^{\text{I}}$ is the indirect genetic effect of group member ${\text{j}}$ of pig ${\text{i}}$. Predictive performance of indirect genetic effects alone was not assessed, since this is not informative for investigating whether indirect genetic effects improve the total predictive ability. A Hotelling-Williams t-test [42, 43] was used to determine whether differences in predictive performances of genetic levels were statistically significant.

For both INDIRECT_GEN and CLASSIC_GEN, genetic effects were predicted 20 times with different weights on ${\mathbf{G}}$ when constructing ${\mathbf{H}}$ (Eq. (4)). The weight on ${\mathbf{G}}$ ranged from 5 to 99% with an interval of 5% except between ${\text{w}} = 95{\text{\% }}$ and ${\text{w}} = 99{\text{\% }}$; i.e. ${\text{w}} \in \left\{ { 5{\text{\% }}, 10{\text{\% }}, 15{\text{\% }}, \ldots ,95{\text{\% }}, 99{\text{\% }}} \right\}$ (Eq. (1)). The purpose was twofold: (1) to find the weight on ${\mathbf{G}}$ that returned the best predictive performance and (2) to examine the ranking between predictive performances of ${\mathbf{DGE}}$ and ${\mathbf{TGE}}$ at different weights on ${\mathbf{G}}$. For both INDIRECT_GEN and CLASSIC_GEN, the genomic analysis with the best predictive performance was used to represent the predictive performance of that model.

The prediction biases of genetic levels were defined as the linear regression coefficient of corrected phenotypes on genetic levels. Just as the predictive performance, the prediction bias was calculated with different weights on ${\mathbf{G}}$.

## Results

### Estimated variance components

Direct genetic variances were similar between CLASSIC and INDIRECT ($\sigma^{2} _{\text{a}_{\text{D}} }$: 3339 ± 450 vs. 3349 ± 451, Table 3). The genetic correlation between direct and indirect genetic effects was not statistically significantly different from zero ($\rho_{{{\text{a}}_{{{\text{D}},{\text{I}}}} }}$: 0.06 ± 0.18). The indirect litter and indirect animal variances were larger for CLASSIC than for INDIRECT ($\sigma _{{{\text{l}}_{\text{I}} }}^{2}$: 37.7 ± 11.1 vs. 19.1 ± 12.2; $\sigma _{{{\text{e}}_{\text{I}} }}^{2}$: 112.9 ± 18.1 vs. 97.4 ± 21.8). The direct heritability was 0.19 ± 0.02 for both CLASSIC and INDIRECT. For INDIRECT, the total heritability was larger than the direct heritability (0.35 ± 0.09 vs. 0.19 ± 0.02).

**Table 3:** Estimates of variance components, heritability, and likelihood ratio statistics with associated p-values

	Classic	Indirect
$\sigma_{{a_{D} }}^{2}$	3339 (± 450)	3349 (± 451)
$\sigma_{{a_{I} }}^{2}$		34.4 (± 16.3)
$\rho_{{a_{D,I} }}$		0.06 (± 0.18)
$\sigma_{u}^{2}$	951 (± 319)	908 (± 306)
$\sigma_{{l_{D} }}^{2}$	636 (± 131)	633 (± 131)
$\sigma_{{e_{D} }}^{2}$	10,971 (± 289)	10,989 (± 295)
$\sigma_{{l_{I} }}^{2}$	37.7 (± 11.1)	19.1 (± 12.2)
$\sigma_{{e_{I} }}^{2}$	112.9 (± 18.1)	97.4 (± 21.8)
$\sigma_{p}^{2}$	17,188 (± 420)	17,597 (± 495)
$\sigma _{TBV}^{2}$		6201 (± 1694)
$h^{2}$	0.19 (± 0.02)	0.19 (± 0.02)
$T^{2}$		0.35(± 0.09)
$- 2LogL$	119,074.46	119,067.12
$P\left( {\Delta LogL} \right)$		0.025

$\sigma _{{{\text{a}}_{\text{D}} }}^{2}$, direct genetic variance. $\sigma _{{{\text{a}}_{\text{I}} }}^{2}$, indirect genetic variance. $\rho_{{{\text{a}}_{{{\text{D}},{\text{I}}}} }}$, genetic correlation between direct- and indirect- genetic effects. $\sigma_{\text{u}}^{2}$, sex-year-month variance. $\sigma _{{{\text{l}}_{\text{D}} }}^{2}$, litter variance. $\sigma_{{{\text{e}}_{\text{D}} }}^{2}$, residual variance. $\sigma_{{1{\text{I}}}}^{2}$, indirect litter variance. $\sigma _{{{\text{e}}_{\text{I}} }}^{2}$, indirect animal variance. $\sigma_{p}^{2}$, total phenotypic variance. $\sigma _{\text{TBV}}^{2}$, total heritable variance. ${\text{h}}^{2}$, direct heritability. ${\text{T}}^{2}$: total heritability. $- 2LogL$, -2 times the log-likelihood. $P\left( {\Delta LogL} \right)$, Chi square p-value (2 degrees of freedom) of log-likelihood ratio between INDIRECT and CLASSIC

### Predictive performance

**Fig. 1:**
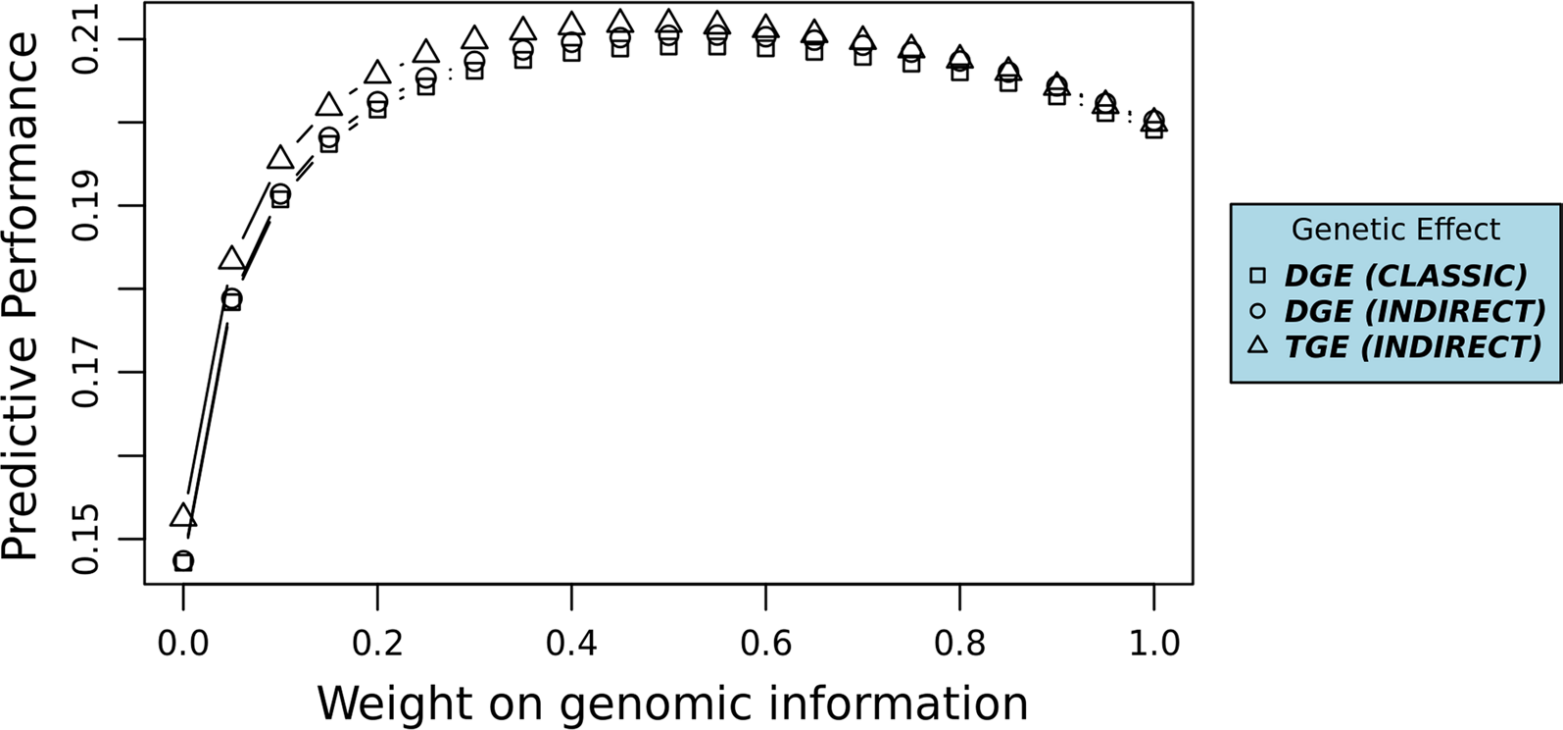
Predictive performances of genetic levels at different weights on genomic information. DGE, Predictive performance of direct genetic effects; TGE, combined predictive performance of direct and indirect genetic effects

The predictive performance of direct genetic effect (DGE) from CLASSIC, DGE from INDIRECT, and total genetic effects (TGE) from INDIRECT were all improved when predicting genetic effects with the single-step relationship matrix rather than the numerator relationship matrix (Table 4 and Fig. 1). The best predictive performance was achieved with TGE from INDIRECT and a 50% weight on the genomic relationship matrix (Fig. 1). The predictive performance of TGE was better than the predictive performance of DGE (Table 4 and Fig. 1), although differences in predictive performance between TGE and DGE were not statistically significant (Table 4). The difference in predictive performance between TGE and DGE decreased as the weight on genomic information increased (Fig. 1; Eq. (1)).

**Table 4:** Predictive performances of direct genetic effects (DGE) and total genetic effects (TGE)

	Predictive performance	
	DGE	TGE
CLASSIC_PED	14.7%^a^	
CLASSIC_GEN	20.9%^b^	
INDIRECT_PED	14.7%^a^	15.3%^a^
INDIRECT_GEN	21.0%^c^	21.2^bc^

Predictive performances with different superscripted letters a, b and c were statistically different according to the Hotelling-Williams t-test

### Prediction bias

**Fig. 2:**
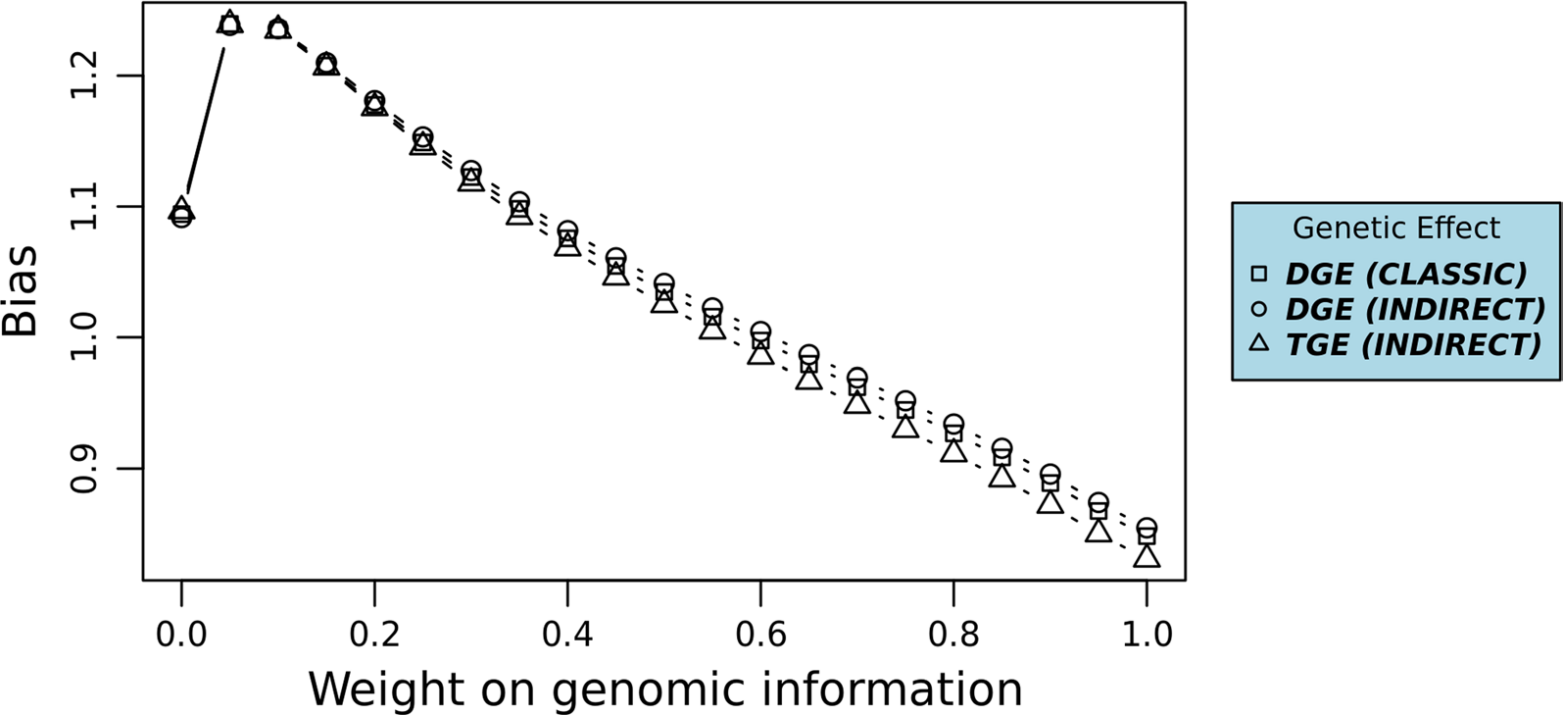
Prediction biases for genetic levels at different weights on genomic information. DGE, Prediction bias of direct genetic effects; TGE, combined prediction bias of direct and indirect genetic effects

The patterns and levels of prediction biases were similar for DGE and TGE (Fig. 2). Prediction bias was lowest with 45% weight on genomic information for DGE and with 50% weight on genomic information for TGE.

## Discussion

As expected, the predictive performances of the models with genomic information were superior to those using pedigree information only (Table 4 and Fig. 1). Thereby, the predictive performance of the indirect genetic model was improved with genomic information. This is in accordance with the previous studies on genomic prediction of TGE by Alemu, et al. [28] and Brinker, et al. [44].

As expected, the predictive performance of TGE was better than the predictive performance of DGE for indirect genetic models. However, the predictive superiority of TGE compared to DGE decreased as the weight on genomic information increased. Although it is difficult to interpret patterns of differences in correlations, this result indicates that indirect genetic effects estimated with genomic information contribute less than indirect effects estimated with pedigree information to the predictive performance of TGE. Therefore, one may speculate that pedigree information explains indirect genetic effects more accurately than genomic information.

Corrected phenotypes were defined as the difference between the raw phenotypes and the expected levels based on fixed effects from INDIRECT. This gives INDIRECT models an advantage over DIRECT models in the prediction analysis. Furthermore, corrected phenotypes can also be calculated as the difference between raw phenotypes and expected levels based on both fixed effects and selected random effects. The correction of phenotypes for random effects relies on the assumption of independence between the random effect used for correction and the genetic effects. If this assumption is violated, the choice of method for calculation of corrected phenotypes can affect the ranking of predictive performances of genetic levels. In this study, we found that the method for calculating the corrected phenotypes influenced the results on predictive performance; i.e. the predictive performance of TGE was inferior to the predictive performance of DGE when correcting the phenotype for both fixed effects and random sex-year-month (results not presented). This susceptibility to method for correction of phenotypes indicates that sex-year-month effects and indirect genetic effects were not independently distributed as assumed by the animal model.

In this study, we attempted to correct for indirect fixed effects. For example, the deviation in weight at test start from the average starting weight of the group (${\text{sWghtDev}}$; see "Methods") is both a direct and indirect covariate. Since ${\text{sWghtDev}}$ was positively associated with later growth rate (results not shown), its direct interpretation is that pigs that are larger than their group members at test start grow faster themselves. However, the sum of the deviations from the mean is zero, and therefore $w_{i} - \bar{w} = - \left( {\mathop \sum \nolimits_{j \ne i} w_{j} - \bar{w}} \right)$; i.e. the effect of ${\text{sWghtDev}}$ is equivalent to the effect of the sum of the group members’ ${\text{sWghtDev}}$. Thereby, the indirect interpretation of ${\text{sWghtDev}}$ is that pigs that weigh less than the average value for the group make their group members grow faster.

It is important to account for both direct and indirect fixed effects when predicting indirect genetic effects [16]. An indirect genetic model can be perceived as a composite animal model that comprises a classical direct animal model component and an indirect animal model component, where each component contains fixed effects, random environmental effects, and random genetic effects. Many previous studies on indirect genetic effects sparsely correct for indirect fixed effects. This may be partly due to very strong confounding between levels of effects for the direct and indirect components in some cases, e.g. in our study, year of birth was strongly confounded with year of birth of group mates. However, indirect fixed effects and direct fixed effects are not necessarily confounded and including indirect fixed effects in the animal model may lead to improved statistical modelling and predictive performance of indirect genetic models.

Similarly to the correction for indirect fixed effects, correction for indirect random effects may affect the predictive performance of animal models. In this study, the only indirect environmental random effect was an indirect environmental animal effect; i.e. an indirect analogue of the direct residual. Most previous studies on indirect genetic effects on the growth rate of finishers have included a random group effect [3, 5, 9, 45], which is equivalent to the indirect environmental animal effect in this study when group sizes are constant. Other studies have examined other indirect effects in addition to the random group effect. For example, Canario et al. [10] and Canario et al. [46] examined the indirect effect of litter, and Nielsen et al. [9] examined the indirect effect of sex. For Canario et al. [46], including indirect litter effects in the animal model reduced the estimated indirect genetic variance by two-thirds. This could indicate that indirect genetic effects capture indirect environmental effects if these are unaccounted for in the animal model. Thus, indirect genetic variances estimated by Arango et al. [3], Duijvesteijn et al. [5], Nielsen et al. [9], Chen et al. [45] may be overestimated due to insufficient correction for indirect environmental effects other than the group effects.

The animal models of this study corrected for both indirect environmental animal effects and indirect litter effects. Nevertheless, the estimated indirect genetic variance component from this study was larger than most previously published indirect genetic variances for finisher growth (34.4 ± 16.3 vs 6–20) [3, 5, 9, 10, 45]. This may be because our dataset only contains phenotypes from a single herd and thereby a narrower distribution of environmental effects than other studies. This is supported by the fact that we obtained larger estimates of direct genetic variances than Nielsen et al. [9], who analyzed finisher growth across more herds in the same population.

Most of the estimated variance components are of similar magnitude to those from other studies. For example, the direct genetic variance (3349 ± 451) is marginally greater than those published by other studies (2762, 2521, and 3200) [9, 47–49]; the direct litter variance (633 ± 131) is in the range of those published by other studies (984, 652, and 1576) [9, 47, 48]; and the residual variance (10,989 ± 295) was greater than those published by other studies (7246, 5439, and 10,000) [9, 47–49]. We may have obtained greater estimates of residual variances because of how we modelled contemporary groups (HYM). Other studies included contemporary groups as fixed effects, while we included contemporary group as a random effect.

In our study, we assumed that indirect genetic effects were not affected by the number of pigs in the group, and not affected by whether pigs were removed from the pen prior to the end of its testing period. It has been suggested that the indirect genetic effect of a pig should be scaled (diluted) based on the number of pigs in its group or the number of days it was in the pen [50, 51]. In our study, dilution according to group size was not relevant since there was only little variation in group size (9 vs. 10). Dilution of indirect genetic effects according to the time the pig spends in the pen was investigated; however, in accordance with Ask et al. [47], dilution did not provide an increase in predictive performance, and was therefore omitted.

In our study, the effects of sex, group size, and stable are confounded. Consequently, INDIRECT assumes that all genetic (co)variance components were equal across sex to avoid confusing the effect of sex with genome by environment interactions when interpreting the results. However, Nielsen et al. [9] found that the genetic correlation between indirect genetic effects of boars and gilts was less than 1 and that both direct and indirect genetic variances differ between boars and gilts. We could not test this in our study due to confounding of sex with stable. In future studies, it would be preferable to use data and animal models that enable the estimation of genetic variance components both within and across boars and gilts.

As mentioned above, based on the results on predictive performance it can be speculated whether indirect genetic effects could be more appropriately modelled by the numerator relationship matrix than the single-step relationship matrix. Thus, we examined an alternative to INDIRECT. In this animal model, direct and indirect genetic effects were uncorrelated; both direct and indirect genetic effects were modelled using single-step relationship matrices; and the weight on genomic information in the single-step matrix ranged from 0 to 99% for indirect genetic effects, but was fixed at 1% for direct genetic effects. The predictive performance of TGE from this alternative model was, surprisingly, superior to any other model developed during our study when indirect genetic effects were modelled with pedigree information only. Meanwhile, the predictive performance of DGE from this model did not depend on the weight on genomic information for indirect genetic effects. We do not recommend the use of this model for prediction as we can not explain this pattern and because the direct genetic effects no longer provide information on indirect genetic effects through their covariance structure. Nevertheless, the pattern of prediction of this alternative model may further indicate challenges with prediction of indirect genetic effects with genomic information. Further research is needed to investigate whether results are replicable both within other populations and with other data to assess the consistency of the results.

Our results both support and challenge prediction of genetic effects with indirect genetic models and genomic information. On one hand, both TGE and DGE from the indirect genetic models with genomic information were superior to DGE from the classical animal models with genomic information. On the other hand, the conclusions are affected by both how heavily genomic information is weighted and the choice of method for calculating corrected phenotypes. We believe that especially the latter indicates insufficient correction for both fixed and random environmental effects. The animal models of this study corrected for environmental effects similarly to previous studies [5, 9, 52]. Therefore, we are not sure that indirect genetic models with genomic informations are ready for implementation in an industrial genetic evaluation system for pigs. Instead, we strongly encourage future studies to attempt to replicate these results in other populations, and to expand the search for indirect environmental effects beyond those that have previously been associated with the trait in a classical genetic model with only direct genetic effects.

## Conclusions

This study provides evidence that: (1) the combined predictive performance of indirect genetic effects and direct genetic effects is superior to the predictive performance of only direct effects; and (2) prediction of genetic levels is more accurate with genomic relationships than with pedigree relationships. The better predictive performance of models with genomic relationships is primarily due to better predictive performance of direct genetic effects. However, it is important to note that the first conclusion was sensitive towards both the definition of corrected phenotypes and the relative weight on genomic information in the single-step relationship matrix.

## Data Availability

The data analyzed during this study is not publicly available as it is owned by SEGES P/S.
